# Comparative Genomics of Environmental and Clinical *Burkholderia cenocepacia* Strains Closely Related to the Highly Transmissible Epidemic ET12 Lineage

**DOI:** 10.3389/fmicb.2018.00383

**Published:** 2018-03-06

**Authors:** Josselin Bodilis, Elodie Denet, Elisabeth Brothier, Arnault Graindorge, Sabine Favre-Bonté, Sylvie Nazaret

**Affiliations:** ^1^Research Group on Environmental Multi-Resistance and Efflux Pump, INRA 1418, UMR CNRS 5557, Laboratoire Ecologie Microbienne, Ecole Nationale Vétérinaire de Lyon, Université de Lyon 1, Villeurbanne, France; ^2^EA 4312 Laboratoire de Microbiologie Signaux et Microenvironnement, Université de Rouen, Mont-Saint-Aignan, France

**Keywords:** *Burkholderia cenocepacia*, Burkina Faso, virulence factors, RND pumps, prophages, antibiotic resistance

## Abstract

The *Burkholderia cenocepacia* epidemic ET12 lineage belongs to the genomovar IIIA including the reference strain J2315, a highly transmissible epidemic *B. cenocepacia* lineage. Members of this lineage are able to cause lung infections in immunocompromised and cystic fibrosis patients. In this study, we describe the genome of F01, an environmental *B. cenocepacia* strain isolated from soil in Burkina Faso that is, to our knowledge, the most closely related strain to this epidemic lineage. A comparative genomic analysis was performed on this new isolate, in association with five clinical and one environmental *B. cenocepacia* strains whose genomes were previously sequenced. Antibiotic resistances, virulence phenotype, and genomic contents were compared and discussed with an emphasis on virulent and antibiotic determinants. Surprisingly, no significant differences in antibiotic resistance and virulence were found between clinical and environmental strains, while the most important genomic differences were related to the number of prophages identified in their genomes. The ET12 lineage strains showed a noticeable greater number of prophages (partial or full-length), especially compared to the phylogenetically related environmental F01 strain (i.e., 5–6 and 3 prophages, respectively). Data obtained suggest possible involvements of prophages in the clinical success of opportunistic pathogens.

## Introduction

The *Burkholderia cepacia* complex (Bcc) is a group of opportunistic pathogens that cause lung infections in immunocompromised and cystic fibrosis (CF) patients. The Bcc (originally described as *Pseudomonas cepacia*) comprises 20 taxonomically related species frequently isolated in water or soil, including plant-associated environments (Ramette et al., 2005; De Smet et al., 2015). *Burkholderia multivorans* and *B. cenocepacia* (formerly Genomovar II and III, respectively) are the most clinically frequent members of Bcc, representing from 50% up to 80% of the infections caused by this complex (Mahenthiralingam et al., 2002; Holden et al., 2009; Peeters et al., 2017). Unlike other opportunistic pathogens isolated from CF patients (e.g., *Haemophilus influenzae* or *P. aeruginosa*), members of the Bcc could lead to the “*cepacia* syndrome.” This syndrome is characterized by a rapid clinical deterioration due to necrotizing pneumonia or sepsis, resulting in early death (Isles et al., 1984; Gibson et al., 2003). Moreover, several Bcc species have been shown to be transmissible among CF patients and are able to cause epidemic outbreaks (Biddick et al., 2003).

Given that isolate can result of variable virulence traits depending on the infected patient (Isles et al., 1984; Govan et al., 1993), the determinants of the *cepacia* syndrome are complex. Indeed, many virulence factors have been highlighted in *B. cenocepacia* (Drevinek and Mahenthiralingam, 2010; Leitao et al., 2010; Bazzini et al., 2011a; Schwager et al., 2013). Briefly, expression of the cable pilus (*cblA*), as well as a 22 kDa adhesion, are required for the initial adhesion of the bacteria to the surface of lung epithelial cells (McClean and Callaghan, 2009). Moreover, a functional flagella and lipase are necessary for cell invasion (Tomich et al., 2002; Mullen et al., 2007), and several other virulence factors enhance the *B. cenocepacia* pathogenicity, like LPS, metalloprotease, siderophores or secretion systems (Leitao et al., 2010; McKeon et al., 2010). Cell-cell communication by quorum sensing (QS), have been described in *B. cenocepacia* to coordinate expression of these different virulence factors, most of them being necessary for the virulence and also for the normal physiology of *B. cenocepacia* (Leitao et al., 2010).

Clinical strains of Bcc exhibit high levels of antibiotic resistance that participates to the mortality of the CF patients (Foweraker, 2009). Multiple mechanisms were described as responsible for this drug resistance, including the production of modifying or degrading enzymes, antibiotic target alterations, cell permeability, and drug efflux. In addition, Bcc strains are able to form biofilm on various biotic and abiotic surfaces, increasing their drug tolerance (Coenye, 2010). Because high level of resistance to most of antibiotics (β-lactams, polymyxins, and aminoglycosides) is now commonly found in Bcc strains, treatment of *B. cenocepacia* by triple antibiotic combination has been proposed (Aaron et al., 2000). Among the antibiotic resistance mechanisms of greatest concern, the RND (Resistance-Nodulation-Cell Division) pumps are particularly problematic because they catalyze the active efflux of many chemically different molecules and, consequently, participate in the multi-drug resistance (MDR) phenotype (Li et al., 2015). No fewer than 15 RND efflux pumps have been described in *B. cenocepacia* J2315, and their roles in antibiotic resistance was demonstrated for at least 5 of them (Bazzini et al., 2011b; Buroni et al., 2014).

DNA–DNA hybridization and phylogenetic analysis of the *recA* gene revealed the presence of four distinct *recA* lineages within *B. cenocepacia*, i.e., genomovar IIIA to IIID (Vandamme et al., 2003). Even though no clear distinction has been established between clinical and environmental Bcc strains on the basis of phenotypic or genotypic criteria (Bevivino et al., 2002; Peeters et al., 2017), *B. cenocepacia* strains isolated from CF patients seem to be more invasive and more virulent than environmental strains (Pirone et al., 2008; Bevivino et al., 2012). Moreover, *B. cenocepacia* IIIA strains were rarely found outside of human infections in comparison to other *B. cenocepacia* and the Bcc (Baldwin et al., 2007). In particular, the *B. cenocepacia* epidemic ET12 lineage, which belongs to the genomovar IIIA and includes the reference strain J2315 (also known as LMG 16656T), is a highly transmissible epidemic *B. cenocepacia* lineage that emerged from the Canada and spread to Europe during the 1990s (Holden et al., 2009). In this study, we describe the genome of F01, a new environmental *B. cenocepacia* strain isolated from soil in Burkina Faso. This environmental strain presents the same *recA* sequence as J2315, and is, to our knowledge, the most closely related environmental strain to the epidemic ET12 lineage. A comparative genomic analysis was performed on the genome of this environmental strain, together with available genomes of five clinical and one environmental *B. cenocepacia* (i.e., J2315, BC7, K56-2, H111, MCO-3, and AU1054 strains). Phylogenetic relationships between these seven *B. cenocepacia* strains were investigated. In addition, antibiotic resistances, virulence phenotype and genomic contents were compared and discussed.

## Materials and Methods

### Bacterial Strains and Sampling Sites

The *B. cenocepacia* strain F01 was isolated form an agricultural soil in the periphery of Ouagadougou in Burkina Faso (sub-sahelian climate). The soil was sampled in a field planted with sorghum in June 2008, 6 months after the crop harvest (Youenou et al., 2016). Characterization of the strain F01 according to the Bcc was performed as described in Mahenthiralingam et al. (2000) by amplification of 1043 bp of the *recA* gene with BCR1 and BCR2 primers.

Six previously sequenced *B. cenocepacia* strains from clinical and environmental origins were included in the study (**Table 1**). The genomes were obtained from NCBI website^1^ and the strains were kindly provided by Dr. Goldberg and Dr. Varga. Briefly, the *B. cenocepacia* stains J2315 (Holden et al., 2009), BC7 and K56-2 (Varga et al., 2013) are members of the highly transmissible genomovar IIIA (ET12 lineage) and were all isolated from CF patients. The *B. cenocepacia* strain H111 is also a member of the genomovar IIIA isolated from CF patient, but this strain is not included in the ET12 lineage (Carlier et al., 2014). The *B. cenocepacia* strain AU1054 is a member of the genomovar IIIB isolated from a CF patient (Chen et al., 2001). The *B. cenocepacia* strain MCO-3 is a member of the genomovar IIIB isolated from the rhizosphere of maize (A. Baldwin, unpublished).

**Table 1:** General genomic features of the studied *B. cenocepacia* strains.

	F01	J2315	BC7	K56-2	H111	MC0-3	AU 1054
Source	Soil	Clinical (CF isolate)	Clinical (CF isolate)	Clinical (CF isolate)	Clinical (CF isolate)	maize rhizosphere	Clinical (CF)
Location	Burkina Faso (Ouagadougou)	United Kingdom (Edinburgh)	Canada	Canada	Germany (Hannover)	United States (Michigan)	United States
Reference	This study	Holden et al., 2009	Varga et al., 2013	Varga et al., 2013	Carlier et al., 2014	Unpublished	Unpublished
Genbank Accesion Number		GCA000009485.1	ALIZ00000000.2	ASM98130.1	ASM23621.4	ASM1950.1	ASM1408.1
Genome size (bp)	8,041,553	8,055,782	7,991,017	7,752,055	7,684,399	7,971,389	7,279,116
# Chromosomes	NA	3	NA	NA	NA	3	3
# Plasmids	NA	1	NA	NA	NA	0	0
# Contigs	91	NA	295	19	71	NA	NA
G+C (%)	67.2	66.9	66.8	67.0	67.4	66.6	66.9
Average CDS length (bp)	906.5	931.0	910,3	938,6	953,5	935.7	945.9
Coding density (%)	88.7	86.9	87,4	87,7	88,2	87.7	87.5
# CDSs	8,185	7,871	7,846	7,391	7,204	7,697	6,961
# Ribosomal RNA operons	NA	6	NA	NA	NA	6	6
# Transfer RNA genes	59	74	69	67	59	67	67
# Putative prophage islands (# full-length propages)	3 (2)	6 (4)	6 (4)	5 (3)	3 (3)	4 (1)	1 (1)
# Strain-specific CDSs	1463	139	391	144	599	1176	760

### Genome Sequencing and Assembly

The draft genome of the strain F01 was performed by the DTAMB/Biofidal structure at the University of Lyon (France) using a Roche 454 GS Junior sequencer (454 Life Sciences, Branford, CT, United States) combined with an Illumina MiSeq approach (Illumina, San Diego, CA, United States). The 454 run led to 143,478 reads with an average read length of 597 bp. The Illumina MiSeq PE 2 × 300 led to 4,946,130 paired-end reads with a mean length of about 195 bp after trimming, a final coverage of × 73 being generated (**Table 1**). Reads were *de novo* assembled using the SPAdes version 3.10.1 (Bankevich et al., 2012). The contigs were reordered according to the genome sequence of the reference strain J2315 using the Mauve Contig Mover (Rissman et al., 2009) of the MAUVE software (Darling et al., 2004). The contigs that could not be replaced according to the J2315 genome sequence were placed at the end of the alignment.

The nucleotide sequences of the strain F01 was deposited into the European Nucleotide Archive^2^ with the accession number ERS2025400 (the study accession is PRJEB23651).

### Genome Annotation and Comparative Genomics

Coding sequences (CDSs) predictions, as well as automatic and manual sequence annotations, were performed using the MicroScope platform pipeline at Genoscope (Vallenet et al., 2013). CDSs were predicted using AMIGene software (Bocs et al., 2003). Automatic functional annotation of the predicted CDSs was performed using the tools integrated in the MicroScope platform (Vallenet et al., 2009). Gene predicted to be involved in functions of interest were manually investigated by using the “genome browser” tool of the platform. Genomic islands and regions of genomic plasticity (RGPs) of each genome were identified using the “RGP finder” tool included in the MicroScope platform by searching for synteny breaks and HGT (horizontally transferred genes) features (Vernikos and Parkhill, 2006; Waack et al., 2006). Antibiotic resistance genes (ARGs), including intrinsic, mutation-driven and acquired resistance, were searched using the CARD database version 1.1.2 (Comprehensive Antibiotic Resistance Database; McArthur et al., 2013) included in the MicroScope platform. Prophage sequences were identified using the PHASTER tool (Arndt et al., 2016) and manually confirmed by eliminating false positive “incomplete prophages” (e.g., transposons). In addition, clustered regularly interspaced short palindromic repeats (CRISPRs) were investigated using the CRISPR Finder tool (Grissa et al., 2007).

### Phylogenetic Analysis

The evolutionary relationships among the seven studied *B. cenocepacia* strains were determined from a concatenated alignment of the orthologous protein sequences of the core genome of these seven strains. Orthologous proteins were identified from bidirectional best-hit BLASTP searches of each strain proteome against J2315’s proteome with an *e*-value parameter threshold of 10e^-5^. Customized computer scripts were then used to extract the best reciprocal hits from all the strains and to align these protein sequences with Clustal omega (Sievers et al., 2011). The alignments were then filtered using Gblocks version 0.91 b (Talavera and Castresana, 2007) with default options and concatenated. A final alignment of 4,555 concatenated proteins (1,514,675 amino acids) was used for the phylogenetic analyses. A phylogenetic tree was reconstructed with the maximum-likelihood method by implementation in RAxML V7.9.5 (Stamatakis, 2006) with 1,000 bootstraps replicates. The phylogenetic tree root was confirmed to be positioned between the two genomovar IIIA and IIIB by reiterating the same protocol with 29 additional *Burkholderia* spp. genomes (data not shown).

Multilocus sequence typing (MLST) of the seven studied strains was performed using seven standard housekeeping genes (*atpD*, *gltB*, *gyrB*, *lepA*, *phaC*, *trpB*, and *recA*) according to the protocol and primers specified in a public database of MLST sequence data^3^ (Spilker et al., 2009). The sequences were obtained from the sequenced genomes.

### Nitrate Respiration Test

Nitrate anaerobic respiration was evaluated using the API 20NE kit (bioMérieux, Marcy-l’Étoile, France), according to the manufacturer’s recommendations.

### Antibiotic Resistance Test

Antibiotic resistances of the strain F01, as well as the six previously sequenced *B. cenocepacia* strains included in our study were determined by the agar diffusion method using a set of 16 antibiotic disks (Bio-Rad, Marnes-la-Coquett, France). The antibiotics tested corresponded essentially to the officially recommended drugs for use in human medicine against infections caused by non-fermenting gram negative bacilli, in particularly against *B. cepacia*: Ticarcillin (TIC, 75 μg), Ticarcillin + clavulanic acid (TCC, 75 + 10 μg), Piperacillin + tazobactam (PPT, 75 + 10 μg), Ceftazidime (CAZ, 30 μg), Cefepime (FEP, 30 μg), Cefpirome (CPO, 30 μg), Chloramphenicol (CHL, 30 μg), Imipenem (IPM, 10 μg), Meropenem (MEM, 10 μg), Ofloxacin (OFX, 5 μg), Levofloxacin (LVX, 5 μg), Ciprofloxacin (CIP, 5 μg), Pefloxacin (PEF, 5 μg), Minocycline (MNO, 30 μg), Colistin (CST, 50 μg), Trimethoprim + sulfamethoxazole (SXT, 23.75 + 1.25 μg). After 24 h at 35°C, the strains were classified as susceptible, intermediate or resistant according to French national guidelines^4^. *P. aeruginosa* CIP 76110 (ATCC 27853) was used as control.

### Virulence Assays

Virulence was determined using the *Dictyostelium discoïdeum* social amoeba, as previously described (Froquet et al., 2009). Strains of *P. aeruginosa* PT5 and *Klebsiella pneumoniae* KpGe were used as negative and positive control for each assay. The axenic *D. discoïdeum* strain AX2 (kindly provided by Anne Vianney, CIRI, University Lyon1) was used for virulence assays. Overnight bacterial culture was diluted in Luria–Bertani (LB) medium to reach an OD_600__nm_ of 1.5. Then, 1 mL of each bacterial suspension was plated on SM Agar (Formedium, Hunstanton, United Kingdom) medium. Plates were dried for 1 h to obtain a dry bacterial layer. Meanwhile, cells of *D. discoïdeum* were washed two times in PAS buffer (Page’s Amoeba Saline buffer; 2.5 mM KH_2_PO_4_, 4 mM MgSO_4_, 0.5 mM CaCl_2_, 2.5 mM, NA_2_HPO_4_, 0.05 mM (NH4)_2_FeII(SO4)_2_) by centrifugation at 1000 × *g* for 10 min. Suspension of amoebae was then adjusted to 2 × 10^6^ cells per ml. This suspension was diluted in series until a final density of about 7800 cells per ml. Five microliters of each dilution were spotted on bacterial lawns. Plates were incubated at 22.5°C during 5 days. After incubation, appearance of phagocytic plaques was evaluated according three categories (Adamek et al., 2011): (i) non-virulent (less than 400 amoebae for lysis plaque formation), (ii) low-virulent (400–2500 amoebae for lysis plaque formation) and (iii) virulent (more than 2500 amoebae). Data shown are the means of two independent experiments, each performed in triplicate

## Results

### Core Genome Phylogeny and Multilocus Sequence Typing (MLST)

**FIGURE 1:**
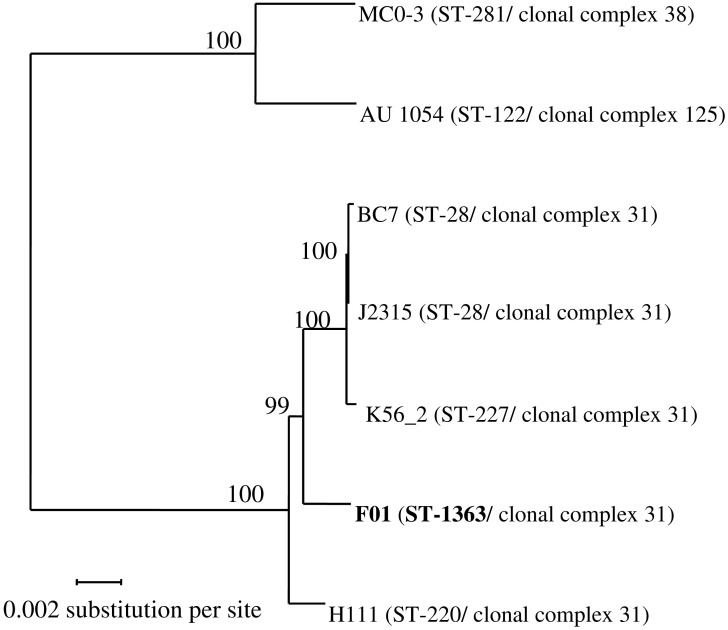
Phylogenetic tree from maximum-likelihood analysis of the core-genome alignments of seven *Burkholderia cenocepacia* strains. In total, 4,555 orthologous proteins were concatenated in an alignment of 1,514,675 amino acids. Bootstraps are indicated in each node. ST, Sequence Type (unique combination of 7 alleles; see Materials and Methods). Two strains belong to the same clonal complex if they share at least four identical alleles.

Sequencing of the *recA* gene of the strain F01 showed the same sequence as the *B. cenocepacia* stains J2315. Using reciprocal BLASTP, protein-coding genes having a 1:1 orthologous relationship to each other were identified across the seven studied *B. cenocepacia*. A total of 4,555 CDSs were identified which could be considered as the core genome for those strains. A final alignment of 1,514,675 amino acids was used for the phylogenetic tree reconstruction by maximum-likelihood method (**Figure 1**). Interestingly this phylogenetic tree is almost fully resolved, with bootstrap values ≥ 99%. Both strains belonging to genomovar IIIB (strains MC0-3 and AU 1054), as defined from the *recA* sequence, were clearly separated from strains belonging to genomovar IIIA. Moreover, the three strains belonging to the epidemic ET12 lineage (strains J2315, BC7 and K56-2), as defined by multilocus enzyme electrophoresis, grouped together. Interestingly, our new environmental F01 strain was closer to the ET12 lineage than the clinical strain H111.

To further investigate these evolutionary relationships, a MLST analysis was performed (**Figure 1**). In this study, all genomovar IIIA strains belong to the same clonal complex (i.e., ≥4 alleles out of 7 are identical), while the two strains of genomovar IIIB belong to two different clonal complexes. In addition, all strains showed a different sequence type (ST), except the strains J2315 and BC7, which belongs to the ST-28. The ST of the F01 strain is new (ST-1363) because of a new allele for the *atpD* gene (*atpD*-437).

### General Features of the F01 Genome and Comparative Genomics

The draft genome of F01 consisted of 8,041,553 bp organized in 91 contigs of size greater than 200 bp (**Table 1**). Therefore, F01 has a genome size close to the other *B. cenocepacia* strains, i.e., between 7,991,017 bp (for BC7) and 8,055,782 (for J2315). Moreover, the average GC content of the F01 genome was 67.2%, that is consistent with the average GC content of the other *B. cenocepacia* (66.9% ± 0.3; **Table 1**). The sequencing methods used in our study (Roche 454 and Illumina MiSeq) did not allow performing the complete genome assembly, because reads were too short to overcome some repeat regions. Thus, the number of chromosomes and presence of plasmids could not be determined in F01, as well as in the other draft genomes available (**Table 1**). Nevertheless, the “Genome Browser interface” available in the Microscope platform^5^) allowed to visualize a global synteny in F01 for the three chromosomes as well as for the plasmid of J2315 (data not shown). This synteny was also conserved in the other *B. cenocepacia* strains studied except for the plasmid that was not detected in the strains H111, MC0-3 and AU 1054.

In total, 8,185 predicted protein-CDSs were identified in the F01 genome that is noticeably higher than the average number of CDSs in the other *B. cenocepacia* strains (7495 bp ± 370). Another difference was the number of tRNAs, which was lower in F01 and H111, compared to the other strains (59 vs 67–74), although this last observation is questionable because of the sequencing technique used which may underestimate the number of repeated genes. Concerning the general genomic features, the most difference was the number of partial or full-length prophages detected (**Table 1**). Indeed, the ET12 lineage strains showed a higher number of (partial or full-length) prophages, with 5–6 prophages detected against 1–4 for the other *B. cenocepacia* strains. More generally, the number of full-length prophages (i.e., potentially functional prophages) seemed associated with the phylogenetic relationships, with 2–4 full-length prophages in genomovar IIIA and only one in genomovar IIIB. However, in genomovar IIIA, the environmental F01 strain presented the lowest number of full-length prophages (**Table 1**). In addition, no CRISPR system were detected in the seven *B. cenocepacia* genomes studied.

Comparative genomics by reciprocal Blast was then performed. Beside the core genome of 4,555 CDS that were used for phylogenetic analysis (**Figure 1**), particular attention was given to variable genes. First of all, we observed that the number of strain-specific genes varied between strains (**Table 1**). The low number of strain-specific genes observed in the ET12 lineage (from 139 to 391 genes) was probably due to the close phylogenetic relationships between the three strains J2315, BC7 and K56-2 (**Figure 1**). However, the highest number of strain-specific genes in the two environmental strains F01 and MC0-3 (1463 and 1176 genes, respectively) could not be phylogenetically explained, when compared to the clinical strains H111 and AU 1054 (599 and 760 genes, respectively), and could be associated with their environmental life style. Annotation of strain-specific genes mainly corresponded to proteins of unknown function (65.0% ± 13.3, compared to 16.7% for the core genome). Despite this lack of annotation, and beside the genes associated with phages, transposition or expression regulation, we focused on two gene categories. On the one hand, several genes specific to F01 were described as involved in xenobiotic degradation, in particular monoxygenases, dioxygenases, cyclases or cytochromes encoding genes, potentially responsible for the degradation of RDX (i.e., Hexahydro-1,3,5-trinitro-1,3,5-triazine) and chlorophenolic compounds (e.g., F01_v2_460557, F01_v2_460615 to F01_v2_460617). On the other hand, analysis of putative metabolic functions revealed more putative genes involved in nitrate respiration, compared to the other *B. cenocepacia* strains. However, although nitrate respiration was phenotypically confirmed by API gallery in F01, this feature was also found among some of our studied *B. cenocepacia* strains (e.g., J2315 and MC0-3).

Variable genes among our seven strains studied were also analyzed by two additional approaches. On one hand, a genomic comparison was performed between the two environmental strains (F01 and MC0-3) and the five other clinical strains. On the other hand, a comparison limited to the genomovar IIIA was performed between F01, H111 and the ET12 lineage strains (J2315, BC7 and K56-2). (i) The first comparison showed only eight genes present in all the clinical strains of *B. cenocepacia* and absent in the two environmental strains. Among these eight genes, the only functionally characterized gene (BCAM2106 in J2315) encodes a non-heme chloroperoxidase, associated with response to oxidative stress. Conversely, 23 genes specific to environmental strains and absent in clinical strains were also highlighted. These genes corresponded to a prophage partially conserved between the two environmental strains and absent in clinical strains (**Table 2**). (ii) The second comparison revealed 745 genes specific to the ET12 lineage strains and absent in the other genomovar IIIA strains. Most of these genes (62.1%) encoded putative proteins of unknown function, while best described genes corresponded to transposons, expression regulation genes and the four prophages specific to the ET12 lineage (**Table 2**).

**Table 2:** Putative prophage islands (PI) identified in the studied *B. cenocepacia* strains.

Prophage-like island^a^	Strain	ORFs	Size (kb)	# ORFS	Homolog prophage among the other studied strains (% of conserved genes)^b^	Closest homolog with previously descripted phage (% of conserved genes)	Description^c^
PI_J2315_1	J2315	BCAL0081-BCAL0107	24.9	27	PI_BC7_4 (100%)	phiE202 (50.0%)	Putative prophage
PI_J2315_2	J2315	BCAL1559-BCAL1606	37.3	49	PI_BC7_6 (100%)	KS10 (100%)	**Prophage** (Goudie et al., 2008)
PI_J2315_3	J2315	BCAM1024-BCAM1096	46.8	76	PI_BC7_2 (100%)	SEN34 (26.3%)	Putative prophage
PI_J2315_4	J2315	BCAM0001AM_1973-BCAM0001AM_2038	46.3	71	PI_BC7_3 (100%)	No homolog	Prophage-like
PI_J2315_5	J2315	BCAS0504-BCAS0554	37.2	52	PI_BC7_5 (100%)	BcepMu (98.1%)	**Prophage** (Summer et al., 2004)
PI_J2315_6	J2315	BCAL2961-BCAL2972	13.9	20	PI_BC7_1 (100%)	No homolog	Prophage-like
PI_BC7_1	BC7	ALIZ_v1_240021-ALIZ_v1_240040	13.9	20	PI_J2315_6 (100%)	No homolog	Prophage-like
PI_BC7_2	BC7	ALIZ_v1_310024-ALIZ_v1_310099	46.8	76	PI_J2315_3 (100%)	SEN34 (26.3%)	Putative prophage
PI_BC7_3	BC7	ALIZ_v1_870050-ALIZ_v1_1440021	46.3	71	PI_J2315_4 (100%)	No homolog	Prophage-like
PI_BC7_4	BC7	ALIZ_v1_1010011-ALIZ_v1_1550014	24.9	27	PI_J2315_1 (100%)	phiE202 (50.0%)	Putative prophage
PI_BC7_5	BC7	ALIZ_v1_1830002-ALIZ_v1_1830053	37.2	52	PI_J2315_5 (100%)	BcepMu (98.1%)	**Prophage** (Summer et al., 2004)
PI_BC7_6	BC7	ALIZ_v1_2510102-ALIZ_v1_2510150	37.3	49	PI_J2315_2 (100%)	KS10 (100%)	**Prophage** (Goudie et al., 2008)
PI_K56-2_1	K56-2	ALJA_v1_70123-ALJA_v1_70152	24.9	27	PI_J2315_1 (100%)	phiE202 (50.5%)	Putative prophage
PI_K56-2_2	K56-2	ALJA_v1_80531-ALJA_v1_80608	44.0	78	PI_J2315_3 (98.6%)	SEN34 (25.6%)	Putative prophage
PI_K56-2_3	K56-2	ALJA_v1_81440-ALJA_v1_81501	45.8	62	PI_J2315_4 (87.3%)	No homolog	Prophage-like
PI_K56-2_4	K56-2	ALJA_v1_120326-ALJA_v1_120375	37.3	49	PI_J2315_2 (98%)	KS10 (100%)	**Prophage** (Goudie et al., 2008)
PI_K56-2_5	K56-2	ALJA_v1_150265-ALJA_v1_150285	13.9	21	PI_J2315_6 (95.2%)	No homolog	Prophage-like
PI_F01_1	F01	F01_v2_10038-F01_v2_10052	13.4	15	PI_MC0-3_1 (20.0%)	phiE125 (7.0%)	Prophage-like
PI_F01_2	F01	F01_v2_50119-F01_v2_50190	68.9	72	PI_MC0-3_1 (41.7%)	KL3 (48.6%)	Putative prophage
PI_F01_3	F01	F01_v2_230115-F01_v2_230168	42.8	56	No homolog	BcepC6B (71.4%)	Putative prophage
PI_H111_1	H111	CAFQ_v1_150037-CAFQ_v1_150122	36.0	85	No homolog	JBD44 (16.4%)	Putative prophage
PI_H111_2	H111	CAFQ_v1_320028-CAFQ_v1_320058	38.8	31	PI_J2315_1 (83.9%)	KL3 (48.1%)	Putative prophage
PI_H111_3	H111	CAFQ_v1_430147-CAFQ_v1_430193	10.8	47	No homolog	phiH111-1	**Prophage** (Lynch et al., 2013b)
PI_MC0-3_1	MC0-3	Bcenmc03_0165-Bcenmc03_0214	38.8	50	PI_F01_2 (41.7%)	KS5 (70.0%)	Putative prophage
PI_MC0-3_2	MC0-3	Bcenmc03_1281-Bcenmc03_1291	10.8	11	No homolog	phi2 (7.7%)	Prophage-like
PI_MC0-3_3	MC0-3	Bcenmc03_6783-Bcenmc03_6787	10.0	12	No homolog	No homolog	Prophage-like
PI_MC0-3_4	MC0-3	Bcenmc03_4251-Bcenmc03_4256	4.3	8	No homolog	No homolog	Prophage-like
PI_AU1054_1	AU 1054	Bcen_4855-Bcen_4870	12.2	19	No homolog	No homolog	Prophage-like

^a^*Prophage and prophage-like regions inferred have been designated “PI_strain_1,” “PI_strain_2,” etc., with “PI” for Prophage Island. ^*b*^For each PI, only the closest homolog was shown. All these homologs were syntenic, expect PI_F01_1 and PI_MC0-3_1. ^*c*^Putative prophage and Prophage-like correspond to PI marked as “complete” and “incomplete,” using PASTER software, respectively (Arndt et al., 2016). PI noted as “Prophage” was experimentally demonstrated*.

### Resistances to Antibiotics

Resistance phenotypes across 16 antibiotics and combinations were investigated using the disk diffusion method (**Table 3**). Five studied *B. cenocepacia* strains (J2315, K56-2, F01, H111 and MC0-3) showed a similar number of antibiotic resistances, with resistance to about half of the tested antibiotics. Indeed, all these strains were resistant to ticarcillin (alone or in combination with clavulanic acid), cefpirome, colistin, and ofloxacin. These same strains were susceptible to ceftazidime (a third generation cephalosporin), chloramphenicol, and minocycline (**Table 3**). Interestingly, no significant differences were found between the antibiotic resistance profiles of clinical (J2315, K56-2, and H111) and environmental (F01 and MC0-3) strains. The BC7 strain was the most susceptible of the strains tested, particularly against fluoroquinolones with susceptibility to ciprofloxacin and pefloxacin. The AU1054 strain was the most resistant of the *B. cenocepacia* strains studied, with a resistance to 13 out of the 16 antibiotics tested. Notably, AU1054 is resistant to ceftazidime, chloramphenicol, and the combination of trimethoprim and sulfamethoxazole (**Table 3**).

**Table 3:** Antibiotic resistances of seven studied *B. cenocepacia* strains.

Drug	Class	J2315	BC7	K56-2	F01	H111	MC0-3	AU1054
Piperacillin + tazobactam	Penicillin	S^a^	S	I	S	S	R	S
Ticarcillin	Penicillin	R	R	R	R	R	R	R
Ticarcillin + clavulanic acid	Penicillin	R	R	R	R	R	R	R
Imipenem	Carbapenem	R	R	R	R	R	S	R
Meropenem	Carbapenem	S	I	I	S	I	S	S
Cefepime	Cephalosporin	S	S	R	S	R	R	R
Cefpirome	Cephalosporin	R	R	R	R	R	R	R
Ceftazidime	Cephalosporin	S	S	S	S	S	S	R
Chloramphenicol	Phenicol	S	S	S	S	S	S	R
Minocycline	Tetracylcine	S	S	S	S	S	S	S
Colistin	Polymyxin	R	R	R	R	R	R	R
Tripmethoprim + sulfamethoxazole	Sulfamide-Trimethoprim	I	S	S	S	S	S	R
Ciprofloxacin	Fluoroquinolone	R	S	R	R	R	R	R
Levofloxacin	Fluoroquinolone	I	S	R	I	S	I	R
Ofloxacin	Fluoroquinolone	R	R	R	R	R	R	R
Pefloxacin	Fluoroquinolone	R	S	I	R	I	R	R

^a^*Interpretations were made according to the recommendations of the antibiogram committee of the French society of microbiology. R, resistant; I, intermediate; S, susceptible*.

In addition to the phenotypic assays, the comprehensive antibiotic resistance database (CARD), including resistance genes and mutations conferring antibiotic resistance, was used to predict the resistome of the strains studied (**Supplementary Table S1**). The resolution of the CARD database ontology is often at the antibiotic class level, which does not allow a direct comparison with resistance phenotypes. In addition, it is well known that ARGs must be expressed to provide effective resistance. Indeed, our genomic resistome analysis suggested potential resistance to all classes of antibiotics, even for strains revealing a susceptible phenotype (**Supplementary Table S1**). Differences observed between resistomes concern only genes encoding efflux pumps, notably the resistance nodulation cell-division (RND) pumps, as well as a specific allele of the *katA* gene involved in the resistance to isoniazid. Indeed, while all genomes contain both *katA* and *katB* genes, potentially conferring resistance to isoniazid, only *katB* was found in the F01 genome (**Supplementary Table S1**).

Because many RND efflux pumps involved in antibiotic resistance have already been described in *B. cenocepacia*, the genes encoding these pumps were exhaustively researched among the strains studied (**Supplementary Table S3**). 16 CDSs annotated as probably encoding 15 RND efflux pumps (HAE-1 and HME families) were found in J2315 (including a heterotrimeric RND pump). A role in antibiotic resistance was demonstrated for at least five of them (Bazzini et al., 2011b). In all the studied *B. cenocepacia*, only 13 of these 16 CDSs were highlighted, including the five efflux pumps involved in antibiotic resistance (**Supplementary Table S3**). The RND-12 pump, involved in copper efflux (HME family), is absent in H111, while the RND-15 pump, whose function is unknown, is absent in the two strains of genomovar IIIB (MC0-3 and AU1054), as confirmed by synteny study (**Supplementary Table S3**). Interestingly, genes potentially encoding the same RND pumps (HAE-1 and HME families) were found in the three strains of the ET12 lineage as well as in the environmental F01 strain (**Supplementary Table S3**). In H111, additional genes encoding for two other HAE-1 efflux pumps, and potentially involved in antibiotic resistance, were found. This strain therefore has genes encoding 16 potential RND pumps. In the two genomovar IIIB strains (MC0-3 and AU1054), additional genes encoding for three other efflux pumps were found, including a HAE-1 efflux pump homologous (orthologous) to one of the additional H111 pumps. Moreover, one of the genes encoding efflux pump is a pseudogene (Bcen_3622, RND-9). MC0-3 therefore has genes encoding 17 potential RND pumps.

### Virulence

**FIGURE 2:**
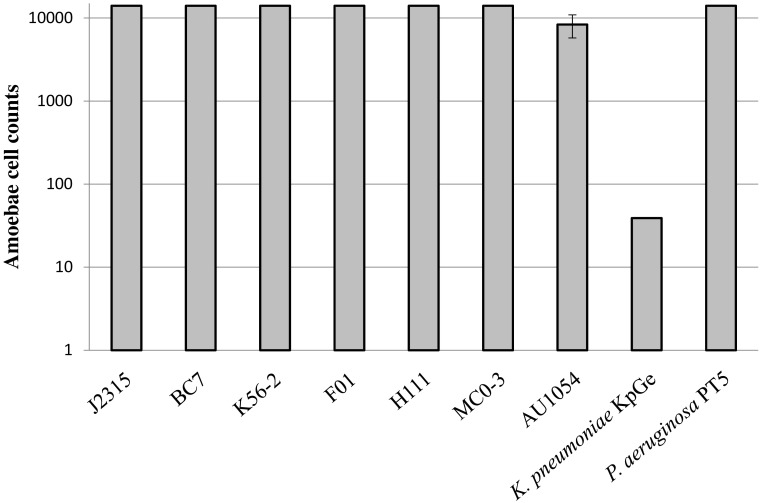
Virulence of *B. cenocepacia* strains determined using the social amoeba *Dictyostelium discoïdeum*. Strains of *Pseudomonas aeruginosa PT5* and *Klebsiella pneumoniae* KpGe were used as positive and negative control, respectively. The axenic *D. discoïdeum* strain AX2 was used for virulence assays. Dilutions of amoebae were spotted on bacterial lawn plates and incubated during 5 days (*n* = 6).

The virulence phenotype of the seven strains of interest was determined by using the *D. discoïdeum* model (Froquet et al., 2009). This assay measures the lowest number of amoeba cells required for effective bacterial predation. The higher the number of amoebae required for bacterial killing, the higher is the ability of bacteria to resist predation. Our results revealed that all *B. cenocepacia* strains can be considered as virulent, compared to the *P. aeruginosa* PT5 positive control (**Figure 2**). Indeed, these strains were able to resist predation of the maximum amount of amoebae tested, excepted for AU1054 which displayed a slightly less virulent phenotype compared to the other *B. cenocepacia* strains. This assay did not seem to display any significant difference of virulence between clinical and environmental strains (**Figure 2**).

Additionally, our analysis of the literature currently available on *B. cenocepacia* virulence genes (Aubert et al., 2008; Drevinek and Mahenthiralingam, 2010) highlighted that almost all virulence genes identified in members of the epidemiologic ET12 lineage are also present in the environmental strain F01, including genes encoding the cable pilus, previously described as ET12 lineage-specific (McClean and Callaghan, 2009) (**Supplementary Table S2**). Only the genes *katA* and *wbcE* (annotated as glycosyl transferase, and involved in lipopolysaccharide synthesis) were absent in the F01 genome. Finally, genes encoding proteins involved in QS, synthesis of cable pilus, iron acquisition or rhamnolipid synthesis were absent in the genomovar IIIB strains, as well as in the H111 strain (**Supplementary Table S2**).

## Discussion

*B. cenocepacia*, especially strains from the ET12 lineage, are among the most prevalent Bcc members that colonize CF patients (Isles et al., 1984; Speert et al., 2002; Mahenthiralingam et al., 2005; Drevinek and Mahenthiralingam, 2010). However, despite that many virulence factors were described, the epidemiological success of some lineages is still misunderstood (Holden et al., 2009; McClean and Callaghan, 2009; Bazzini et al., 2011a; Gonyar et al., 2015). The *B. cenocepacia* species is characterized by a large variety of ecological niches, from patient-associated to environmental niches. Although some studies have shown that clinical strains of *B. cenocepacia* have a higher virulence than environmental strains (Pirone et al., 2008; Bevivino et al., 2012), the distinction between clinical and environmental strains based on phenotypic or genotypic criteria remains difficult (Bevivino et al., 2002; Peeters et al., 2017). Indeed, to our knowledge, strains belonging to the ET12 lineage had not yet been isolated from a non-clinical environment (Baldwin et al., 2007). Thus, this is the first time that an environmental strain phylogenetically related to the ET12 lineage, is described. The environmental F01 strain belongs to the same clonal complex and possess, to date, the phylogenetically closest genome to ET12 genomes, by comparison to other available *B. cenocepacia* genomes (**Figure 1**). In this context, this study provides a new evolutionary aspect to understanding the emergence of the ET12 lineage, and more generally, the epidemic risk represented by opportunistic pathogens.

The presence of genes involved in xenobiotic degradation and nitrate respiration suggested that F01 is well adapted to agricultural soil. Historically, F01 was isolated in 2008 from a soil characterized by an absence of amendment for at least 10 years (Youenou et al., 2016). A recent anthropogenic origin of F01 could therefore be excluded. Moreover, it is important to notice that another strain with a similar *recA* sequence (also similar to J2315 *recA* sequence) was isolated 3 years later from the same site (data not shown). Although we cannot affirm that this isolate is a direct descendant to F01, this strain displayed specific traits in common with F01 (i.e., same virulence phenotype, ability to metabolize nitrate, same pattern of antibiotic resistances) (data not shown). Taken together, these two isolates show that strains adapted to agricultural soils can be phylogenetically related to the ET12 epidemic lineage.

This observation raises the question of the specific pathogenic properties of strains involved in clinical infections, particularly when these strains have been described as opportunistic pathogens. So, among phylogenically related strains why some displayed an efficient success in clinical situations and not the others? Beyond the capacity for infection, are there specific properties of opportunistic pathogenic strains that explain their epidemiological success? In an attempt to answer these questions, we conducted in this present work a comparative study between ET12 strains and our new environmental strain of *B. cenocepacia*, focusing on two parameters that characterize clinical strains: antibiotic resistances and virulence.

Surprisingly, no significant differences in antibiotic resistance were found between ET12 strains and F01, as well as between clinical and environmental strains of genomovar IIIB (**Table 3**). As previously observed, the numerous RND pumps in *B. cenocepacia* are likely to represent the main genetic determinants responsible for the MDR phenotype frequently described in this bacterial species (Holden et al., 2009; Perrin et al., 2010; Bazzini et al., 2011b; Buroni et al., 2014). Interestingly, although this cannot be correlated with the phenotypes observed, some variation in the number of RND efflux pump genes between the *B. cenocepacia* strains were observed, while the other ARGs are almost all conserved in this bacterial species (**Supplementary Table S3**). Although the genes coding for RND pumps are generally found as conserved in a given bacterial species, we already identified horizontal gene transfers coding for such efflux pumps in environmental strains of *Stenotrophomonas maltophilia*, potentially associated with an MDR phenotype (Youenou et al., 2015; Bodilis et al., 2016). Because, both organic (e.g., PAHs; Hearn et al., 2003) and inorganic (e.g., metals; Nies, 1995) contaminants, as well as root exudates (Eda et al., 2011), were previously described as inducers and/or substrates of RND efflux pumps, a role of these environmental factors in the selection and dissemination of efflux-mediated resistances is probable (Martinez et al., 2009). Without necessarily directly inducing an increase in antibiotic resistance in these environmental opportunistic pathogenic strains, such factors could at least promote future clinical adaptability by selecting strains with multiple efflux pumps. Indeed, the emergence of multidrug-resistant bacteria is often linked to mutations that overexpress efflux pumps (Li et al., 2015).

As surprising as the results of antibiotic resistance phenotypes, all strains of *B. cenocepacia*, including F01, were virulent, according to our amoeba assay (**Figure 2**). In genomovar IIIB, we observed a probable non-significant slight decrease in virulence for the clinical strain (AU1054) compared to the environmental strain (MC0-3). The phenotypic virulence assay we used was validated by several studies on strains of *B. cenocepacia* or other opportunistic pathogenic species (Aubert et al., 2008; Froquet et al., 2009; Steinert, 2011). Moreover, the conservation of the virulence phenotype that we observed for all the *B. cenocepacia* tested is in agreement with the presence in their genome of most of the genes encoding the virulence factors already described in J2315 (**Supplementary Table S2**). Additional tests on other environmental strains of *Burkholderia* spp. showed virulence traits in all of the *B. cenocepacia* tested, while other *Burkholderia* spp. (e. g., *B. dolosa* and *B. multivorans*) were not virulent (data not shown). Although this should be deeply investigated, the virulence phenotype of *Burkholderia* spp. appears to be species-specific and not related to the clinical origin of the strains. This observation is in connection with other studies showing a significant number of opportunistic pathogenic bacteria in the rhizosphere. This environment could select bacterial virulence properties during interaction with plant roots or during the intense microbial competition present in the rhizosphere (Berg et al., 2005; Mendes et al., 2013). Recently, a study has even shown that a rhizospheric *B. cenocepacia* strain enhances its virulence during mice infection (Bragonzi et al., 2017). Consequently, although several Bcc strains have developed beneficial interactions with their plant hosts, the difficulties of distinguishing between pathogenic and harmless strains prevents the use of Bcc members as biocontrol agents (e.g., Vial et al., 2011).

Beyond the virulence, we also considered the epidemiological properties of ET12 strains, notably the genomic signatures specific to these clinical strains. Thus, Cable A is one of the few virulence factors previously described as specific to ET12 strains (Graindorge et al., 2010). However, we also identified the gene encoding this virulence factor in the genome of the environmental strain F01 (**Supplementary Table S2**), excluding an epidemiological signature associated with this gene. Similarly, the plasmid initially described in J2315 is found in all ET12 strains and in the environmental strain F01. An analysis of the databases revealed that this plasmid is also present in *B. cenocepacia* HI2424 (genomovar IIIB) and other *Burkholderia* species (*B. thailandensis*, *B. gladioli*, and *B. glumae*) (data not shown). Therefore, the occurrence of this plasmid in *Burkholderia* spp. does not appear to be related to particular species, environment, or virulence. Finally, the occurrence of prophages in the genome of ET12 strains appears to be the most interesting epidemiological signature (**Table 2**).

The most important differences, especially in genomovar IIIA, were therefore related to the number of prophages identified in the genomes (**Table 1**). The ET12 lineage strains displayed a noticeable greater number of (partial or full-length) prophages, especially compared to the phylogenetically related environmental F01 strain (i.e., 5–6 and 3 prophages, respectively). However, it is difficult to determine whether these prophages are directly involved into the ecological adaptation or whether they represent only habitat or lifestyle markers. On the one hand, some studies noticed the presence of genes encoding virulence factors into prophages (i.e., Sitkiewicz et al., 2006; Tobe et al., 2006). Several prophages have already been characterized in *B. cenocepacia* (**Table 2**; Summer et al., 2004; Goudie et al., 2008; Lynch et al., 2013b), without the presence of genes encoding new virulence factors detected (Summer et al., 2007). However, given the number of prophages not or only slightly characterized in *B. cenocepacia* (**Table 2**), a direct role of these prophages in the virulence and/or in particular epidemiological properties of the ET12 strains cannot be excluded. On the other hand, it can also be suggested that the substantial presence of prophages in the genome of ET12 strains is not the cause but the consequence of the epidemiological success of these strains. Thus, according to the “kill-the-winner” ecological model, dominant strains in an ecological niche are more likely to be targeted by phage attacks (Rodriguez-Valera et al., 2009). The ET12 strains, due to their epidemiological success, should indeed be at least transiently dominant in their ecological niche. The substantial presence of prophages in their genome would result from the numerous phage attacks that occurred. According to this model, the environmental strain F01 has probably never been so dominant in its telluric environment, which would be in accord with the lowest number of prophages in its genome. Finally, a third hypothesis could explain this variation in the number of prophages. A recent ecological model, “the piggyback-the-winner model,” predicts that phages integrate as prophages and undergo the lysogenic replication cycle instead of the lytic cycle when bacterial density and activity increase, especially in internal mucosal surfaces, such as human lungs (Silveira and Rohwer, 2016).

The dramatic decline in the effectiveness of antibiotics has generated renewed interest in phage therapy (Abedon et al., 2017). The use of bacteriophages as antibacterial therapeutics is especially important for targeting those pathogens for which antibiotic treatments are limited, e.g., in *B. cenocepacia* (Lynch et al., 2013a; Roszniowski et al., 2017). In this context, it is interesting to note that the absence of a self-defense CRISPR system makes these therapeutic approaches even more promising. Finally, the particular occurrence of prophage in the epidemiological ET12 strains reinforces the need to better understand the interactions between *B. cenocepacia* and bacteriophages, particularly in a clinical context.

## Author Contributions

JB performed the bioinformatics analysis presented in the work. ED performed the virulence assays. EB performed the antibiotic resistance tests. All authors contributed to the design and interpretation of the results, as well as to writing the article, and approved it for publication.

## Conflict of Interest Statement

The authors declare that the research was conducted in the absence of any commercial or financial relationships that could be construed as a potential conflict of interest.

## Footnotes

1 http://www.ncbi.nlm.nih.gov/

2 http://www.ebi.ac.uk/ena

3 http://pubmlst.org/bcc/

4 http://www.sfm-microbiologie.org/

5 https://www.genoscope.cns.fr/agc/microscope

## Supplementary Material

TABLE S1 Genomic resistome in seven studied *B. cenocepacia* strains.

TABLE S2 Virulence genes in seven studied *B. cenocepacia* strains.

TABLE S3 RND efflux systems in seven studied *B. cenocepacia* strains.
